# Pooled Analysis of Baricitinib Tolerability in Patients With Atopic Dermatitis in Relation to Acne, Headache, and Gastrointestinal Events From 8 Clinical Trials

**DOI:** 10.1089/derm.2022.0027

**Published:** 2023-07-17

**Authors:** Andreas Wollenberg, Leon Kircik, Eric Simpson, Dennis Brinker, Norito Katoh, Maria Jose Rueda, Maher Issa, Fan Yang, Meghan Feely, Andrew Alexis

**Affiliations:** From the * Department of Dermatology and Allergy University Hospital, Ludwig Maximilian University of Munich, Munich, Germany.; † Department of Dermatology, Free University Brussels, University Hospital Brussels, Brussels, Belgium.; ‡ Icahn School of Medicine at Mount Sinai, New York, New York, USA.; § Oregon Health & Science University, Portland, Oregon, USA.; ‖ Eli Lilly and Company, Indianapolis, Indiana, USA.; ¶ Department of Dermatology Kyoto Prefectural University of Medicine, Kyoto, Japan.; # Icahn School of Medicine, Mount Sinai, New York, New York, USA.; ** Weill Cornell Dermatology, New York, New York, USA.

## Abstract

*****Abstract: Background***:**

Tolerability issues including acne, nausea, and headache have been reported with Janus kinase (JAK) inhibitors for moderate-to-severe atopic dermatitis (AD).

**Objectives:**

To report outcomes of tolerability adverse events (AEs) for baricitinib, a JAK1/JAK2 inhibitor, in patients with moderate-to-severe AD.

**Methods:**

Acne, headache, and gastrointestinal AEs are reported from placebo-controlled and long-term extensions of pooled data in the baricitinib AD clinical trial program. Proportions of patients with AEs, incidence rates (IRs)/100 patient-years at risk, and median time to onset/duration of AEs were calculated.

**Results:**

In 2531 patients treated with baricitinib, most AEs were mild to moderate in severity. Headache was the most common AE of tolerability (median of 14–26 days after first dose of baricitinib, lasting ≤3 days). IRs of acne were <5 in any group lasting up to a median of 90 days with no severe AEs. Diarrhea was the most common gastrointestinal AE, lasting a median of ≤7 days. There were few study drug interruptions (n = 6) and permanent discontinuations (n = 5) for tolerability AEs.

**Conclusions:**

For the AEs of tolerability analyzed, baricitinib appears to be well tolerated. Overall, the frequency of these AEs in patients being treated for moderate-to-severe AD was low with few leading to study drug interruption or permanent discontinuation.

**Clinical Trial Registration number:**

NCT02576938; NCT03334396; NCT03334422; NCT03428100; NCT03435081; NCT03733301; NCT03334435; NCT03559270

## Capsule summary

- In 2531 baricitinib-exposed patients with moderate-to-severe atopic dermatitis, headache was the most common adverse event (AE) of tolerability. Across all AEs, the majority were mild to moderate in severity.
- There were few study drug interruptions (n = 6 in all patients who received baricitinib) or permanent study drug discontinuations (n = 5) due to AEs of tolerability, and baricitinib was generally well tolerated.

Atopic dermatitis (AD) is a chronic, heterogenous, and highly pruritic inflammatory skin disease characterized by skin barrier dysfunction and excessive T cell activation.^1–4^ Clinical signs and symptoms of AD including erythema, dry skin, erosions, excoriations, lichenification, pruritus, sleep disturbance, and pain affect daily quality of life. During treatment for AD, common adverse events (AEs) of tolerability including headache, acne, and gastrointestinal issues in patients taking Janus kinase (JAK) inhibitors can also affect daily quality of life.

Current systemic treatments approved for AD include the interleukin (IL) 4/IL13 inhibitor dupilumab; the IL13 inhibitor tralokinumab, and the JAK inhibitors abrocitinib, upadacitinib, and baricitinib. Assessments of safety outcomes for these treatments have found them to have favorable risk–benefit profiles, however, tolerability-related AEs have been reported with each. For the JAK inhibitors, nausea was the most commonly reported AE with abrocitinib (6.1% and 14.6% in 100- and 200-mg groups, respectively),^5^ whereas with the use of upadacitinib, acne (15.1% and 24.5% in 15- and 30-mg groups, respectively) and headache (8.9% and 8.1% in 15- and 30-mg groups, respectively) were the most commonly reported AEs of tolerability.^6^

Baricitinib, a selective JAK1/JAK2 inhibitor, is approved in several countries for the treatment of moderate-to-severe AD and is recommended in adults who are candidates for systemic therapy.^7^ It is also approved for the treatment of moderate-to-severe rheumatoid arthritis in adults in Europe, Japan, and the United States, and for the treatment of severe alopecia areata in Japan and the United States. In completed phases 2 and 3 clinical trials, baricitinib as monotherapy and in combination with topical corticosteroids improved signs and symptoms of AD.^8–12^

Overall safety with baricitinib has been disclosed,^13,14^ but not as it relates to details of the intensity, evolution, and treatment considerations of headache, acne, and gastrointestinal issues (nausea, vomiting, constipation, abdominal pain, and diarrhea). In this study, we report pooled safety of key AEs of tolerability of baricitinib in patients with moderate-to-severe AD from previously published data of the placebo-controlled phase of clinical trials and long-term extension (LTE) studies in the AD clinical program with exposure up to 2 years.^13^

## METHODS

### Study Designs and Patients

This analysis included patient-level safety data from 6 double-blinded randomized placebo-controlled studies (phase 2: NCT02576938; phase 3: NCT03334396 [BREEZE-AD1], NCT03334422 [BREEZE-AD2], NCT03428100 [BREEZE-AD4], NCT03435081 [BREEZE-AD5], NCT03733301 [BREEZE-AD7]), 1 double-blinded randomized LTE study (NCT03334435 [BREEZE-AD3]), and 1 open-label LTE study (NCT03559270 [BREEZE-AD6]) with data cutoffs of December 13, 2019 (BREEZE-AD3) and December 24, 2019 (BREEZE-AD6) in the ongoing LTE studies.

The study design and patient inclusion/exclusion criteria for each study have been described previously.^8–12^ In brief, patients were ≥18 years old, with moderate-to-severe AD, defined as Eczema Area and Severity Index score of ≥16 (≥12 for the phase 2 study), Validated Investigator Global Assessment-Atopic Dermatitis^®^ score of ≥3 (not used in the phase 2 study), ≥10% body surface area involvement at baseline, inadequate response to topical therapies, and in BREEZE-AD4, inadequate response to cyclosporine.

Key exclusion criteria were concomitant skin conditions that could affect assessment of AD lesions; history of eczema herpeticum within 12 months before screening or ≥2 episodes of eczema herpeticum at any time previously; a venous thromboembolic event (VTE) or major cardiovascular event within 12 weeks of screening or high risk for VTE, the definition of which varied by study: for BREEZE-AD1, BREEZE-AD2, BREEZE-AD4, and BREEZE-AD7, history of recurrent (≥2) VTE or patients were considered at high risk of VTE as deemed by the investigator; for BREEZE-AD5, history of VTE or patients were considered at high risk for VTE as deemed by the investigator, or having 2 or more of the following risk factors for VTE: age >65 years, body mass index >35 kg/m^2^, oral contraceptive use and current smoker; for BREEZE-AD6, history of VTE or patients were considered at high risk of VTE as deemed by the investigator.

Studies were conducted in accordance with the Declaration of Helsinki and good clinical practice guidelines and approved by individual institutional review boards at each participating study center. All patients provided written informed consent.

### Analysis Sets

Outcomes are reported for 3 integrated analysis sets:

1. Placebo-controlled data set: 2-mg and 4-mg baricitinib versus placebo during the 16-week placebo-controlled period for patients in the phase 2 study and 4 phase 3 studies (BREEZE-AD1, BREEZE-AD2, BREEZE-AD4, and BREEZE-AD7).
2. 2 mg to 4 mg extended data set (extended data set): data from the randomized LTE study BREEZE-AD3 and the 16-week placebo-controlled data (including the phase 2 study and BREEZE-AD4). BREEZE-AD3 enrolled patients from originating studies BREEZE-AD1, BREEZE-AD2, and BREEZE-AD7 giving a total exposure to baricitinib up to 105 weeks of treatment.
3. All-bari AD data set (All-bari): data for all patients who received at least 1 dose (1 mg, 2 mg, or 4 mg) of baricitinib from any of the 8 clinical trials at any time.

### Safety Outcomes

Tolerability outcomes included acne, headache, and gastrointestinal AEs (diarrhea, nausea, vomiting, constipation, and abdominal pain) reported as preferred terms based on MedDRA version 22.1. Three preferred terms related to abdominal pain were reported: abdominal pain, abdominal pain upper, and abdominal pain lower. These treatment-emergent AEs, including any that were serious AEs or led to interruption or discontinuation of study drug, were assessed.

### Statistical Analysis

Because the ratio of baricitinib-to-placebo randomization was not the same across all studies, study size adjusted percentages and study exposure time adjusted incidence rates (IRs) per 100 patient-years at risk (PYR) were calculated for AEs to provide adequate direct comparisons between treatment groups for the placebo-controlled and extended data sets. Study size adjusted percentages were derived using study weights based on total sample size per study. Study exposure time adjusted IRs per 100 PYR of observation time, with observation time censored at event date, were derived using study weights based on total patient-years of exposure per study.

For All-bari, IRs were calculated as the number of patients with an AE per 100 PYR of observation time with observation time censored at start of AE. Percent and IRs for All-bari were not adjusted by exposure time or study size due to patients switching from placebo to baricitinib at different times. Percentages of patients with mild, moderate, or severe AEs were calculated and median time to onset of AEs from start of study drug and median duration of AEs were calculated.

## RESULTS

At baseline, disease characteristics and history of prior AD therapies were similar between baricitinib doses and placebo and across all data sets (Table 1). A total of 2531 patients were given ≥1 dose of baricitinib for 2247 patient-years, a median of 310.0 days and 42% of patients had ≥1 year exposure to baricitinib.

**Table 1.** Baseline Demographics and Disease Characteristics

	**Placebo-Controlled (to Week 16)**			**All-bari-AD^a^ (*N* = 2531)**
	**Placebo (*N* = 743)**	**Bari 2 mg (*N* = 576)**	**Bari 4 mg (*N* = 489)**	
Age, years, mean (SD)	35.7 (13.1)	35.9 (13.5)	35.8 (13.2)	36.4 (13.7)
Female, n (%)	298 (40.1)	205 (35.6)	170 (34.8)	994 (39.3)
Duration since diagnosis, years	24.9 (14.6)	25.0 (14.3)	24.7 (14.8)	25.0 (14.9)
Prior topical corticosteroids, n (%)	646 (86.9)	496 (86.1)	430 (87.9)	2255 (89.1)
Prior systemic cyclosporine, n (%)	254 (38.7)	255 (52.8)	172 (41.1)	791 (34.0)
vIGA-AD^®^ of 4, n (%)	324 (46.7)	257 (47.7)	211 (46.8)	1037 (46.9)
EASI, mean (SD)	31.5 (12.7)	31.3 (13.2)	32.1 (12.9)	31.1 (12.8)
SCORAD, mean (SD)	67.8 (13.3)	67.9 (13.4)	68.1 (13.2)	67.5 (13.4)
DLQI, mean (SD)	14.5 (7.7)	14.0 (7.6)	14.0 (7.9)	14.2 (7.7)
Itch NRS, mean (SD)	6.9 (2.0)	6.6 (2.2)	6.7 (2.1)	6.8 (2.1)
Skin pain NRS, mean (SD)	6.3 (2.4)	6.1 (2.5)	5.9 (2.5)	6.1 (2.5)

*AD, atopic dermatitis; bari, baricitinib; DLQI, Dermatology Life Quality Index; EASI, Eczema Area and Severity Index; NRS, numerical rating scale; SCORAD, SCORing Atopic Dermatitis; SD, standard deviation; vIGA-AD, Validated Investigator Global Assessment-Atopic Dermatitis*.

### Tolerability Event Outcomes

Headache was the most common AE of tolerability examined in this analysis across all analysis sets with 3.3% (IR, 11.9) of patients in the placebo group to 6.3% (IR, 21.4) of patients in the 4-mg group reporting the AE during the first 16 weeks of treatment (placebo-controlled period); during the LTE the IR for headache decreased to 7.6 in the All-bari group (Table 2). During the placebo-controlled period, headaches occurred at a median of 14.0 (2-mg group) and 26.0 (4-mg group) days after the first dose of baricitinib and lasted ≤2.0 days (Table 2). In All-bari, the median time to onset of headache was 47.0 days with a median duration of 3.0 days. The majority of headaches (95.6% in All-bari) were mild or moderate in severity, and there were no severe AEs in the placebo-controlled period.

**Table 2.** Frequency of Treatment-Emergent Adverse Events of Tolerability and Severity and Duration of These Events

	**Placebo-Controlled (to Week 16)**			**2 mg to 4 mg Extended**		All-bari-AD^*^
	**Placebo (*N* = 743, PYE = 211.8)**	**Bari 2 mg (*N* = 576, PYE = 169.1)**	**Bari 4 mg (*N* = 489, PYE = 147.1)**	**Bari 2 mg (*N* = 576, PYE = 425.5)**	**Bari 4 mg (*N* = 489, PYE = 459.3)**	**(*N* = 2531, PYE = 2247.4)**
Headache, n (adj %) [adj IR/100 PYR]†	28 (3.3) [11.9]	37 (5.9) [21.1]	35 (6.3) [21.4]	43 (6.9) [10.8]	46 (8.3) [10.6]	166 (6.6) [7.6]
Mild, n (adj%)	19 (2.2)	26 (4.3)	26 (4.7)	31 (5.0)	32 (5.7)	109 (4.3)
Moderate, n (adj%)	9 (1.1)	11 (1.7)	9 (1.6)	12 (1.8)	13 (2.4)	53 (2.1)
Severe, n (adj%)	0	0	0	0	1 (0.2)	4 (0.2)
Median time to onset, days	25.0	14.0	26.0	28.0	38.5	47.0
Median duration, days	2.0	1.0	2.0	1.0	2.0	3.0
Acne, n (adj %) [adj IR/100 PYR]†	7 (0.7) [2.4]	8 (1.2) [4.0]	9 (1.4) [4.7]	15 (2.2) [3.3]	17 (2.7) [3.8]	74 (2.9) [3.3]
Mild, n (adj%)	4 (0.4)	6 (0.9)	9 (1.4)	11 (1.6)	17 (2.7)	61 (2.4)
Moderate, n (adj%)	3 (0.3)	2 (0.3)	0	4 (0.6)	0	13 (0.5)
Severe, n (adj%)	0	0	0	0	0	0
Median time to onset, days	27.0	55.5	38.0	112.0	106.0	146.0
Median duration, days	62.0	22.5	90.0	53.0	90.0	82.5
Gastrointestinal events, n (adj %) [adj IR/100 PYR]†						
Diarrhea, n (adj %) [adj IR]	15 (1.8) [6.2]	10 (1.3) [4.3]	15 (2.7) [9.0]	15 (2.0) [3.1]	19 (3.2) [4.6]	78 (3.1) [3.5]
Mild, n (adj%)	13 (1.6)	10 (1.3)	12 (2.2)	13 (1.7)	15 (2.6)	62 (2.4)
Moderate, n (adj%)	2 (0.2)	0	3 (0.5)	2 (0.3)	4 (0.6)	14 (0.6)
Severe, n (adj%)	0	0	0	0	0	2 (0.1)
Median time to onset, days	16.0	30.0	13.0	71.0	16.0	68.5
Median duration, days	7.0	4.5	3.0	4.0	3.0	3.0
Abdominal pain, n (adj %) [adj IR/100 PYR]	9 (1.2) [4.2]	11 (1.5) [5.0]	7 (1.3) [4.2]	13 (1.9) [2.9]	7 (1.3) [1.8]	32 (1.3) [1.4]
Mild, n (adj%)	4 (0.6)	8 (1.1)	5 (1.0)	10 (1.5)	5 (1.0)	25 (1.0)
Moderate, n (adj%)	4 (0.5)	3 (0.4)	1 (0.1)	3 (0.4)	1 (0.1)	6 (0.2)
Severe, n (adj%)	1 (0.1)	0	1 (0.2)	0	1 (0.2)	1 (0.0)
Median time to onset, days	11.0	27.0	42.0	28.0	42.0	59.5
Median duration, days	4.0	3.0	5.0	3.0	5.0	5.0
Abdominal pain upper, n (adj %) [adj IR/100 PYR]	10 (1.2) [4.1]	10 (1.6) [5.3]	14 (2.5) [8.5]	14 (2.1) [3.2]	15 (2.7) [3.6]	42 (1.7) [1.8]
Mild, n (adj%)	9 (1.1)	9 (1.4)	12 (2.2)	13 (1.9)	12 (2.2)	34 (1.3)
Moderate, n (adj%)	0	1 (0.2)	1 (0.2)	1 (0.2)	2 (0.4)	7 (0.3)
Severe, n (adj%)	1 (0.1)	0	1 (0.2)	0	1 (0.2)	1 (0.0)
Median time to onset, days	6.5	29.0	31.5	49.5	37.0	45.0
Median duration, days	1.0	3.0	9.5	5.5	11.0	6.0
Abdominal pain lower, n (adj %) [adj IR/100 PYR]	0	0	1 (0.2) [0.6]	0	2 (0.4) [0.5]	2 (0.1) [0.1]
Mild, n (adj%)	0	0	1 (0.2)	0	2 (0.4)	2 (0.1)
Moderate, n (adj%)	0	0	0	0	0	0
Severe, n (adj%)	0	0	0	0	0	0
Median time to onset, days	0	0	4.0	0	70.0	70.0
Median duration, days	0	0	19.0	0	13.5	13.5
Nausea, n (adj %) [adj IR/100 PYR]	8 (0.8) [2.7]	14 (1.8) [5.8]	4 (0.8) [2.5]	16 (2.1) [3.4]	8 (1.4) [1.8]	49 (1.9) [2.1]
Mild, n (adj%)	7 (0.7)	12 (1.6)	4 (0.8)	13 (1.9)	8 (1.4)	38 (1.5)
Moderate, n (adj%)	1 (0.1)	2 (0.2)	0	3 (0.3)	0	10 (0.4)
Severe, n (adj%)	0	0	0	0	0	1 (0.0)
Median time to onset, days	18.0	13.0	17.0	17.5	95.0	56.0
Median duration, days	4.5	4.0	3.0	3.0	6.5	5.0
Vomiting, n (adj %) [adj IR/100 PYR]	8 (0.9) [3.1]	5 (0.7) [2.3]	3 (0.6) [1.9]	8 (1.2) [1.7]	3 (0.6) [0.6]	25 (1.0) [1.1]
Mild, n (adj%)	5 (0.5)	3 (0.4)	3 (0.6)	6 (0.9)	3 (0.6)	19 (0.8)
Moderate, n (adj%)	2 (0.3)	2 (0.3)	0	2 (0.3)	0	5 (0.2)
Severe, n (adj%)	1 (0.1)	0	0	0	0	1 (0.0)
Median time to onset, days	13.0	32.0	30.0	76.0	30.0	81.0
Median duration, days	1.5	2.0	2.0	2.0	3.0	2.0
Constipation, n (adj %) [adj IR/100 PYR]	1 (0.1) [0.3]	4 (0.5) [1.7]	1 (0.2) [0.6]	6 (0.7) [1.1]	2 (0.4) [0.4]	22 (0.9) [1.0]
Mild, n (adj%)	1 (0.1)	4 (0.5)	1 (0.2)	6 (0.7)	2 (0.4)	20 (0.8)
Moderate, n (adj%)	0	0	0	0	0	2 (0.1)
Severe, n (adj%)	0	0	0	0	0	0
Median time to onset, days	3.0	21.0	57.0	43.5	173.5	61.0
Median duration, days	4.0	36.0	1.0	53.5	135.5	72.5

^*^ *All-bari AD includes bari 1 mg, 2 mg, and 4 mg.*

^†^ *IRs for the placebo-controlled data sets and the 2 mg to 4 mg extended data set are study size adjusted rates; adjusted percentages are only shown for the placebo-controlled data set.*

*AD, atopic dermatitis; adj, adjusted; bari, baricitinib; IR, incidence rate; PYE, patient-years exposure; PYR, patient-years at risk.*

Acne frequencies were low in the placebo-controlled period (<1.5% in any group; IRs <5 in any group) with an IR of 3.3 in the All-bari group. Acne occurred at a median of 38.0 to 146.0 days after first dose of baricitinib and a median of 27.0 days after placebo, lasting up to a median of 90.0 days. The majority of AEs were mild (82.4% in the All-bari group), with no severe AEs in any group (Table 2). In the All-bari group, 13 patients reported 15 AEs of moderate acne. For 1 of the patients with 2 moderate AEs, acne vulgaris of the face and back was reported on the same day. The other patient with 2 moderate AEs reported acne folliculitis 82.0 days after recovering from an acne AE.

At the time of database lock, 10 of these moderate AEs were recovered or recovering and 5 were not recovered. Nine patients received treatment for the AE of acne that included topical adapalene, benzoyl peroxide, and clindamycin and oral nadifloxacin, azelaic acid, ozenoxacin, erythromycin, and zinc. Of the 5 patients whose moderate acne AE was not recovered, 3 patients received treatment that was ongoing at the time of data lock, but 2 of those had only received treatment for 1 day; the remaining 2 patients did not receive treatment for the AE of acne. Of the patients with AE of acne that was recovered/recovering and received treatment, the average duration of treatment ranged from 7 to 251 days.

Diarrhea was the most common gastrointestinal AE, occurring at a median of 13.0 to 71.0 days after start of study drug and lasting a median of 3.0 days (baricitinib 4 mg and All-bari) to 7.0 days (placebo). The majority of AEs (79.5% in the All-bari group) were mild, with only 2 severe AEs, both in the All-bari group (Table 2). No AEs of diarrhea led to discontinuation of study drug. Of the 3 preferred terms for abdominal pain, “abdominal pain, upper” was the most commonly reported, with low frequencies in the placebo-controlled period (≤2.5% in any group with IR of 8.5 in the 4-mg group); in All-bari the IR was 1.8.

Median time to onset ranged from 6.5 days (placebo group) to 49.5 days (baricitinib 2 mg in extended data set), and median duration ranged from 1.0 (placebo group) to 11.0 days (baricitinib 4 mg in extended data set). For the preferred term of abdominal pain, frequencies were ≤1.5% in the placebo-controlled period (IR of 4.2 in the 4-mg group) with an IR of 1.4 in All-bari. Median time to onset ranged from 11.0 (placebo) to 59.5 days (All-bari) and median duration ranged from 3.0 to 5.0 days.

For both preferred terms, the majority of AEs were mild in severity; there was 1 patient in the 4-mg group with a severe AE of abdominal pain and a severe AE of “abdominal pain, upper” (both terms reported on the same day during the placebo-controlled period), 1 patient in the placebo group with a severe AE of abdominal pain, and 1 patient in the placebo group with a severe AE of “abdominal pain, upper.” Only 2 AEs of abdominal pain lower were reported, both were mild, lasting a median of 13.5 days.

Nausea was reported infrequently during the placebo-controlled period (≤1.8% in any group; IR of 5.8 in the 2-mg group) with an IR of 2.1 in All-bari. Median time to onset ranged from 13.0 (baricitinib 2 mg in placebo-controlled data set) to 95.0 (baricitinib 4 mg in the extended data set) days with median duration ranging from 3.0 (baricitinib 4 mg in placebo-controlled and baricitinib 2 mg in the extended data set) to 6.5 days (baricitinib 4 mg in the extended data set). The majority of AEs (78% in All-bari) were mild, with only 1 severe AE in the All-bari group that did not lead to study drug discontinuation.

Frequency of vomiting was low during the placebo-controlled period (<1% in any group; IR of 3.1 in the placebo group) with an IR of 1.1 in All-bari; median time to onset ranged from 13.0 (placebo) to 81.0 days (All-bari) and median duration was ≤3.0 days in all groups. The frequency of constipation during the placebo-controlled period was ≤0.5% in any group (IR of 1.7 in the 2-mg group during placebo-controlled period ) with an IR of 1.0 in All-bari and no severe AEs in any of the groups. Median time to onset ranged from 4.0 (placebo) to 173.5 days (baricitinib 4 mg in the extended data set) and median duration of constipation varied from 1.0 to 135.5 days (baricitinib 4 mg in placebo-controlled and extended data sets, respectively).

No AEs of tolerability in this analysis were serious AEs. Overall, there were few study drug interruptions or permanent discontinuations. In the placebo-controlled period, study drug interruptions were for headache (n = 1, baricitinib 4 mg) and abdominal pain (n = 2, placebo; n = 1, baricitinib 2 mg), and permanent discontinuations were for headache (n = 1, baricitinib 4 mg) and abdominal pain (n = 1, baricitinib 2 mg; n = 1, baricitinib 4 mg). In the All-bari group, study drug interruptions included headache (n = 4), vomiting (n = 1), and abdominal pain (n = 1), and there were permanent discontinuations due to headache (n = 2), nausea (n = 1), and abdominal pain (n = 2).

## DISCUSSION

In this report focusing on AEs of tolerability in patients receiving baricitinib for the treatment of moderate-to-severe AD, the frequency of AEs was low, with very few severe and no serious AEs and most AEs lasting for a short duration. Tolerability in clinical trials has been defined as the degree to which overt AEs can be tolerated by the subject^15^ and these AEs can cause patients to not take a medication as prescribed with treatment interruptions or discontinuation of therapy.

This also holds true for medications with a confirmed efficacy on subjective symptoms such as baricitinib.^16,17^ During the placebo-controlled period in our study, there were only 2 study drug interruptions in either baricitinib group and 3 permanent discontinuations due to AEs of tolerability, similar to reports of other JAK inhibitors.^5,6^

AEs of tolerability have been reported in overall assessments of safety for JAK inhibitors in the treatment of AD, though no serious AEs were reported for these AEs for upadacitinib or abrocitinib. Although head-to-head studies are lacking, the tolerability profile of baricitinib is in line with that reported in trials of other oral JAK inhibitors in the treatment of AD (namely abrocitinib and upadacitinib). Headaches were the most common AEs of tolerability in our study occurring in 6.6% of patients in the All-bari group compared with upadacitinib, which reported 8.9% and 8.1% through week 52 in all patients who received at least 1 dose of 15 and 30 mg, respectively.^6^

During the baricitinib placebo-controlled period, 5.9% and 6.3% of patients in baricitinib 2 mg and 4 mg, respectively, reported headaches, compared with abrocitinib, in which 5.9% and 7.8% were reported in the 100- and 200-mg doses, respectively, during the placebo-controlled period.^5^ Median duration of headaches was slightly less for baricitinib (1.0 and 2.0 days for 2 mg and 4 mg in the placebo-controlled period) compared with 3.5 days and 5.0 days for abrocitinib 100 and 200 mg.

Acne has been reported across JAK inhibitors, but was not as common in our study (1.2% and 1.4% in 2 mg and 4 mg, respectively, through 16 weeks) as in upadacitinib (10.1% and 16.5% in 15 and 30 mg in the 16-week placebo-controlled period)^6^ or abrocitinib (1.6% and 4.7% in 100 and 200 mg in the 16-week placebo-controlled period).^5^ Acne had the longest duration of the tolerability AEs in our study, lasting up to 90 days in the 4-mg group in the placebo-controlled and extended data sets, but none led to interruption or permanent discontinuation of study drug.

We assessed several gastrointestinal AEs from the baricitinib studies, some of which have been captured as common (occurring in ≥2% of patients) treatment-emergent AEs including diarrhea, nausea, and upper abdominal pain,^12^ and others that occur with less frequency (vomiting and constipation). Gastrointestinal AEs have been reported as common AEs for abrocitinib, but not for upadacitinib. Nausea was the most common tolerability AE for abrocitinib occurring in 6.1% and 14.6% in 100- and 200-mg groups, respectively, in the placebo-controlled period^5^ compared with 1.8% in baricitinib 2 mg and 0.8% in baricitinib 4 mg in the placebo-controlled period.

Limitations in our study include that incidence rates provide estimates of the number of patients experiencing an AE per 100 patient-years of exposure and can be viewed relative to IRs from the literature. However, any comparisons are for descriptive context only; inferences cannot be made as study and treatment are confounded and risk over time can change due to reasons other than treatment exposure. In addition, within the clinical trials included in our analysis, the quality of reporting safety events may vary from center to center and from study to study such as issues with coding and terminology of AEs, and data collection was still ongoing at the time of data lock for this analysis, so final outcomes of some AEs are yet to be determined. Other limitations include those similar to observational data.

## CONCLUSION

For the AEs of tolerability analyzed in this study, baricitinib appears to be well tolerated. Based on the entirety of the baricitinib safety data, headache, nausea, abdominal pain, and acne are considered adverse drug reactions. Overall, the frequency of AEs related to tolerability for baricitinib in patients being treated for moderate-to-severe AD was low. Few of these AEs led to temporary interruption or permanent discontinuation of the study drug in patients being treated for moderate-to-severe AD.
